# Deformation of Attractor Landscape via Cholinergic Presynaptic Modulations: A Computational Study Using a Phase Neuron Model

**DOI:** 10.1371/journal.pone.0053854

**Published:** 2013-01-11

**Authors:** Takashi Kanamaru, Hiroshi Fujii, Kazuyuki Aihara

**Affiliations:** 1 Department of Innovative Mechanical Engineering, Kogakuin University, Tokyo, Japan; 2 Department of Intelligent Systems, Kyoto Sangyo University, Kyoto, Japan; 3 Institute of Industrial Science, The University of Tokyo, Tokyo, Japan; University of Jaén, Spain

## Abstract

Corticopetal acetylcholine (ACh) is released transiently from the nucleus basalis of Meynert (NBM) into the cortical layers and is associated with top-down attention. Recent experimental data suggest that this release of ACh disinhibits layer 2/3 pyramidal neurons (PYRs) via muscarinic presynaptic effects on inhibitory synapses. Together with other possible presynaptic cholinergic effects on excitatory synapses, this may result in dynamic and temporal modifications of synapses associated with top-down attention. However, the system-level consequences and cognitive relevance of such disinhibitions are poorly understood. Herein, we propose a theoretical possibility that such transient modifications of connectivity associated with ACh release, in addition to top-down glutamatergic input, may provide a neural mechanism for the temporal reactivation of attractors as neural correlates of memories. With baseline levels of ACh, the brain returns to quasi-attractor states, exhibiting transitive dynamics between several intrinsic internal states. This suggests that top-down attention may cause the attention-induced deformations between two types of attractor landscapes: the quasi-attractor landscape (Q-landscape, present under low-ACh, non-attentional conditions) and the attractor landscape (A-landscape, present under high-ACh, top-down attentional conditions). We present a conceptual computational model based on experimental knowledge of the structure of PYRs and interneurons (INs) in cortical layers 1 and 2/3 and discuss the possible physiological implications of our results.

## Introduction

**Figure 1:**
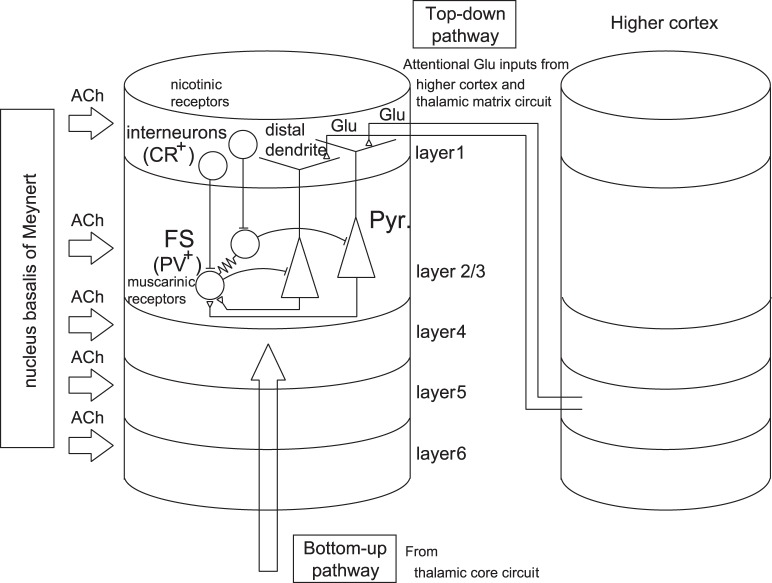
Schematic diagram of cortical layers in the early visual cortex (V1/V2). The bottom-up spike signals via layer 4 from the thalamus core circuit and the top-down spike signals onto layer 1 (and layer 6) interact in the perception of external sensory stimuli in the early cortex (V1/V2). Moreover, acetylcholine (ACh) is transiently released from the nucleus basalis of Meynert to all the layers associated with top-down attention. In layers 2/3, pyramidal neurons (PYRs) that project their apical distal dendrites to layer 1, interneurons (INs), and 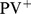 fast spiking neurons exist. Moreover, it is also known that ACh to layer 1 depolarizes calretinin positive (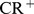) INs in layer 1 through nicotinic receptors. However, we do not consider the latter effect in our model for simplicity.

The perception of external sensory stimuli is an important aspect of cognition, and it has long been a target of neuroscientific research. Perception of external visual stimuli arises from interactions between two streams of signals in the early visual cortex, *i.e.*, the bottom-up spike signals via layer 4 from the thalamus core circuit and the top-down spike signals carrying attention, expectation, and/or contexts onto layers 1 and 6 (see Figure 1).

However, the brain also performs internal processes such as mental imagery or voluntary recall of a scene in episodic memory (see, *e.g.*, [1]–[3]). These instances of visual processing do not involve external stimuli. How does the stream of top-down signals arriving at cortical layer 1 organize dynamical activity to temporarily reconstruct internal representations? This problem brings up a fundamental question about top-down neural processing: what is the nature of the top-down signals that are projected in cortical layer 1?

The cortex exhibits spontaneous activity even in the absence of external stimuli. Possible roles of such spontaneous activity have long been a controversial issue (see, *e.g.*, [4]–[8]). Ongoing and evoked activity in the primary and secondary visual cortices V1 (area 17) and V2 (area 18) of anesthetized cats has been studied by optical imaging, local field potentials and single cell recordings [9]–[13]. In particular, Kenet *et al.* [12] observed intriguing spontaneous activity in V2 of anesthetized cats with both eyes closed [12]. In fact, the activity of the visual cortex with both eyes closed, that is, in the absence of external stimuli, was neither quiet nor random. Instead, it exhibited dynamics that transitively switched between different internal states after a few hundreds of milliseconds. The activity patterns observed under these conditions resembled the orientation selectivity patterns of the visual cortex that are embedded through learning. These results have modified our understanding of cortical dynamics by suggesting that cortical circuits have a number of preexisting and intrinsic internal states. These states are thought to represent stimulus features, and the cortex fluctuates between such intrinsic states in the absence of conscious attention and external inputs (see also [13], [14]).

The mathematical nature of intrinsic internal states such as an orientation selectivity remains controversial. If viewed from the standpoint of conventional static theory, an internal state could be interpreted as a stable equilibrium. That is, the internal state may be viewed as an attractor to which the state of a neural system converges. However, experimental evidence indicates that the system only stays in these states temporarily [12]. After a few hundreds of milliseconds, the system transits to a different internal state. The continuous transitions indicate that internal states are not stable in the classical sense of dynamical systems theory. Therefore, such dynamics could be viewed as an expression of quasi-attractors, a concept in contemporary dynamical systems theory. Roughly, a quasi-attractor is an attractor in that there are positive-measure orbits approaching and temporarily persisting in state space. However, a quasi-attractor may simultaneously possess repelling orbits from itself. The Milnor attractor is a mathematically rigorously defined example of a quasi-attractor, and may thus provide a mechanism for allowing transitions to and from a quasi-attractor (for a detailed definition of the Milnor attractor, see, *e.g.*, [15]). It should be noted that the concept of quasi-attractors may include a wider class of non-classical attractors than the Milnor attractor. However, quasi-attractors have not yet been formalized mathematically in detail. Therefore, in this paper, we use the term quasi-attractor to include possible but unknown classes of non-classical attractors, where classical attractors are usually stable equilibrium points, stable limit cycles, stable quasi-periodic attractors, and low-dimensional chaotic attractors. Quasi-attractors can also be found in the field of chaotic associative memory in neural networks [16]–[25], in which patterns stored in the network become quasi-attractors and the network exhibits transitive dynamics between stored patterns.

What is the neural mechanism underlying the transitive dynamics observed in the visual cortex? More importantly, what are the possible roles of such dynamics in cognitive functions? We have previously presented a working hypothesis focused on these questions [26]–[28]. With top-down attention, whether overt or covert [29], or even in mental imagery [1]–[3], we postulate that two concurrent flows are projected onto the cortex. The first one is a projection of ACh and gamma-aminobutyric acid (GABA) onto all six layers ascending from the nucleus basalis of Meynert (NBM). This transmission is triggered by Glu spikes from the medial prefrontal cortex (mPFC) to the NBM [30]. Moreover, in light of the attention-to-memory (internal representation) hypothesis [31]–[33], we hypothesize that ACh is also released transiently by internal attention and/or expectation. This hypothesis constitutes a cornerstone of the scenario described in our quasi-attractor hypothesis.

Behavioral and immunotoxin studies indicate that ACh is involved in top-down attention. A blockage of ACh from NBM, either as a consequence of disease or drug application, causes a severe loss of selective attention, sustained attention, and divided attention, along with a shift in attention. The death of cholinergic cells in NBM is known to be associated with the Dementia with Lewy bodies (DLB), the most salient symptoms of which are recurrent complex visual hallucinations (RCVHs) [34]. The second attentional signal is Glu spike volleys projecting onto layer 1 from higher cortical areas and the thalamic matrix circuit (see Figure 1). These two concurrent flows (referred to here as attentional flows) are the main components of the top-down mechanism described in the present manuscript. It has been reported that cholinergic afferents have specific synaptic connections with postsynaptic targets, rather than releasing ACh non-specifically [35]. However, the specificity of the corticopetal ACh projections on the cortex remains controversial.

Here, we use the term ongoing state to refer to a cortical dynamical state in which the cortex has no external stimuli and no or minimal top-down attentional flows except intrinsic noise. In the experiment by Kenet *et al.*, this situation was realized by anesthetization and closing the eyes [12]. Our basic proposal is that in the non-attentional ongoing state, the cortex exhibits transitive dynamics among locally existing quasi-attractor states if no external stimuli are applied. We also hypothesize that these transitive dynamics are maintained so long as the ACh concentration remains at a baseline level. In the present manuscript, we aim to understand how top-down signals onto cortical layer 1 organize dynamical activity to reconstruct temporarily internal representations.

Here, we first introduce our quasi-attractor hypothesis. Then we define a single-unit network, which is a building block of a multiunit network for associative memory. By using a multiunit network composed of multiple unit networks with a built-in memory pattern, we show that our quasi-attractor hypothesis is feasible. Lastly, we confirm that our results are consistent with other proposals on the roles of ACh [36], [37].

## Results

### Quasi-attractor Hypothesis

We summarize our scenario as a quasi-attractor hypothesis (see also [26]–[28]).

**Figure 2:**
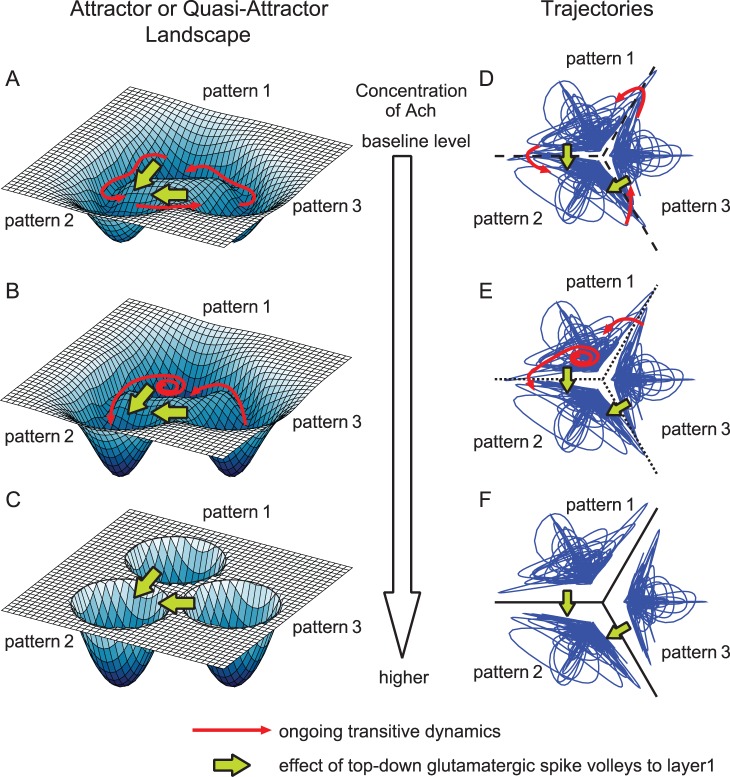
Schematic diagrams of deformation of the attractor landscape induced by release of ACh into cortical layers 2/3, shown in two ways. (A) and (D) Quasi-attractors observed when the concentration of ACh does not exceed its baseline level. In Figure A, the instability of the quasi-attractor is represented by the shallow depth of potential in the landscape, and each quasi-attractor is found to be unstable because there exist repelling orbits from itself. In Figure D, the instability of the quasi-attractor is shown as crossings of trajectories over the boundaries, and each quasi-attractor is unstable because there are many crossing points. The trajectories of the network state successively transit among quasi-attractors as indicated by red arrows. By the top-down Glu spike volleys indicated by green arrows, the network state would jump to another quasi-attractor. However, it would soon transit to other quasi-attractors again. (B) and (E) Quasi-attractors observed when the ACh level is somewhat high. The probability of transitions becomes low, but each quasi-attractor remains unstable. (C) and (F) Stable attractors observed when the concentration of ACh is much higher. Transitive dynamics are not observed because quasi-attractors are stabilized and they become attractors. When top-down Glu spike volleys are injected to cortical layer 1, the trajectories jump to the target pattern (in this example, the attractor of pattern 2) in a short time.

With a low (baseline) ACh level (*i.e.*, in a non-attentional state), patterns associated with memory exhibit the temporal forms of quasi-attractors and transition between quasi-attractors during recall, as indicated by thin red arrows in Figures 2A and 2D. Such dynamics are based on the following four neural mechanisms.

1. In an attentional state in the presence of a relatively high but transient ACh levels due to transient projections from the NBM (see, *e.g.*, [38], [39]), memories are formed as attractors, *i.e.*, the system is in an attractor landscape (A-landscape). An A-landscape refers to the spatial structure of basins of attractors in the state space. Schematic images of attractor states are shown in Figures 2C and 2F.
2. In non-attentional ongoing states, ACh levels return to baseline (see, *e.g.*, [40]) causing attractors become quasi-attractors. Consequently, the attractor landscape becomes a landscape with quasi-attractors (a Q-landscape) as shown in Figures 2A and 2D.
3. The arrival of ACh associated with top-down attention temporarily recovers the A-landscape (see, *e.g.*, [40]). First, the staying time at each quasi-attractor increases as shown in Figures 2B and 2E, and then quasi-attractors become attractors as shown in Figures 2C and 2F.
4. The arrival of increased ACh is also accompanied by top-down spike volleys projected on layer 1. The synapses of these inputs contact with the apical distal dendrites of specific pyramidal neurons (PYRs) in layer 2/3, and enable the orbit (state) to jump into the relevant attractor. These effects are illustrated by the thick green arrows in Figure 2.

The theoretical link that connects transient ACh release to the dynamical event of attractor stabilization lies in the fact that high ACh decreases the inhibition of PYRs by virtue of presynaptic, muscarinic disinhibition. As a result, the inhibitory connections to PYRs are weakened presynaptically. Conversely, when low ACh increases presynaptic inhibition, the inhibitory connections are strengthened and the attractors are destabilized. This is a consequence of a more general principle, which states that inhibition may destabilize attractors under certain conditions [16], [17], [21].

Our hypothesis may help explain why two concurrent signals, *i.e.*, top-down spike volleys onto layer 1, and corticopetal ACh released from NBM, are necessary in the process of voluntary memory recall. At the same time, the distinct nature of these two flows when viewed from the standpoint of dynamical systems will become clear. The former is a type of external force that makes the trajectory of the cortical network jump into the basin of a relevant attractor (memorized pattern), whereas the latter (ACh projection) acts as a bifurcation parameter that changes the attractor landscape.

Our model is based on studies of the ongoing states of V1 and V2 of cats [9]–[11], and it is motivated by the experimental results obtained by Kenet *et al.* [12]. Our aim is to use a computational model to discuss a possible neural mechanism underlying the transitive dynamics together with its possible roles in cognition. We constructed a model of the superficial cortical layers (layers 1 and 2/3) [41] of the early visual cortex (V1/V2). The model is conceptual in that it is a simple coupled system consisting of PYRs and interneurons (INs), presumably parvalbumin-positive (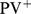) fast spiking (FS) neurons. Together, these cell types constitute the principal neural populations in layer 2/3. Concurrently, our model might reflect the possible influence of ACh on the nonlinear dynamics of neurons (such as oscillatory behavior) in layer 2/3 at least to some extent. We believe that the dynamics generated in our model may apply to many different neural systems as discussed in more detail in the Discussion section.

Chaos is random motion that is generated by a deterministic rule. Chaotic dynamics in neural systems are observed both in single neurons *in vitro* such as the squid giant axon [42], [43] and the Onchidium giant neuron [44], and in models of single neurons [45]–[47]. Chaotic dynamics were also observed in models of pulse-coupled neural networks [48]–[52]. Importantly, modeling studies have suggested that chaotic dynamics are useful in some neural computations such as escaping from local minima in optimization problems and chaotic transitions among memory states in associative memory models [16]–[25], [53], [54].

Our network was composed of phase neuron models (also known as a theta neurons [55]), which model type-I spiking neurons [56], [57]. In our previous work, we constructed a unit (that might be regarded as a minicolumn [58]) composed of coupled excitatory and inhibitory neurons, and we examined the mechanisms governing synchronized firing. In the present simulations, we investigate coupled systems of such units with excitatory and inhibitory interactions. The advantage of using phase neurons is that their Fokker-Planck equation [59] can be numerically analyzed in detail because the phase neuron model is governed by a one-dimensional differential equation. For instance, in a simple network with one unit, we found complex bifurcations of dynamics including chaotic synchronized firing [50] (see below for details). Moreover, since each individual unit in the network is internally synchronized when isolated, coupled units can be mutually synchronized in a periodic manner [60] under some conditions. In some cases, we also observe that units showing chaotic dynamics instead of periodic ones are mutually synchronized after coupling [51]. Further, chaotic associative memory in multiunit networks has been analyzed [61].

### A Network of PYRs and INs (Unit Model)

**Figure 3:**
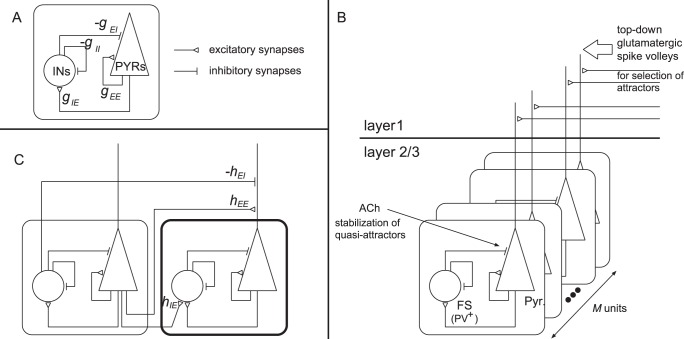
Schematic diagram of our model. (A) As an elemental model of a small network in layers 2/3 in the early visual cortex, we construct a unit model that is composed of 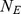 PYRs and 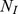 INs, each of which is modeled as a phase neuron connected to all the other neurons globally. We set 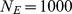 and 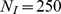 or we take the limit 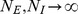 in the analysis of the Fokker-Planck equations (see the Methods section). (B) The multiple units in cortical layers 2/3. ACh decreases inhibitions to PYRs through the presynaptic, muscarinic disinhibitions, and it stabilizes the quasi-attractors. The top-downs Glu spike volleys to the apical distal dendrites of PYRs contribute to the selection of attractors. (C) Connections between two units. Only the connections from the left unit to the right one are shown for simplicity.

**Figure 4:**
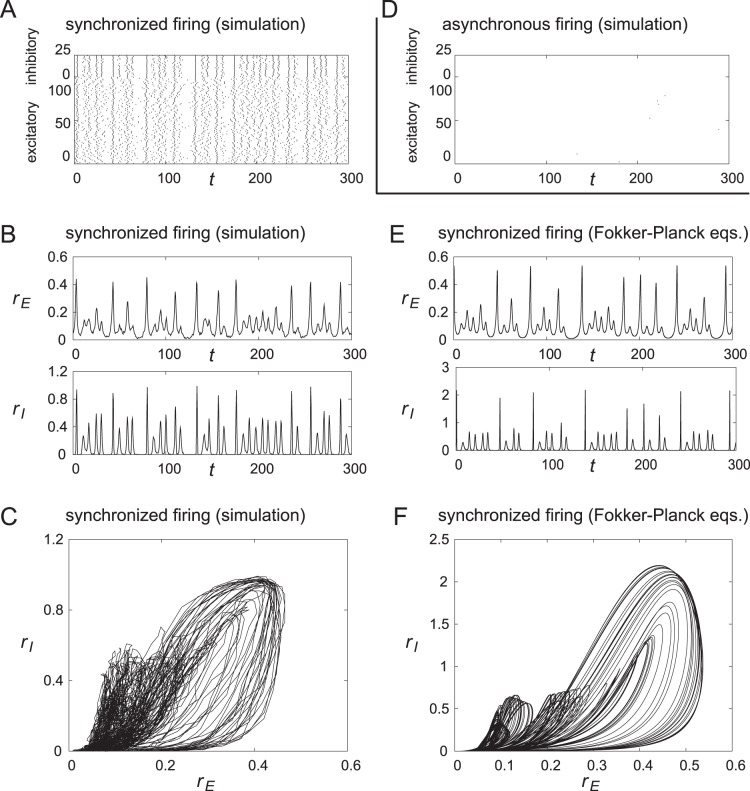
Results of simulations in a unit. (A), (B), and (C) Chaotic synchronization observed in a unit model with 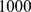 PYRs and 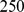 INs. The values of the parameters are 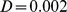, 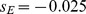, 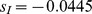, 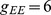, 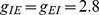, 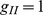, 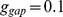, 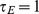, 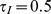, and 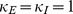. (A) Raster plot of spikes of 100 PYRs and 25 INs (randomly chosen). The firing times of neurons have correlations and we call such firing synchronized. (B) Temporal changes in instantaneous firing rates 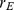 and 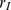 of the excitatory ensemble 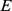 and the inhibitory ensemble 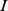, respectively, calculated from the firing in Figure A. It is observed that 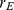 and 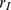 fluctuate, and it is found that this fluctuation is caused by chaotic dynamics and not by a stochastic one. (C) Trajectory in the (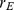, 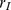) plane obtained from the data in Figure B. It is observed that the trajectory has some complex structure. (D) Asynchronous firing observed in this unit. Raster plot of spikes of 100 PYRs and 25 INs (randomly chosen). The number of firing is very few because the firing rates are low. (E) and (F) Chaotic synchronization in a unit with an infinite number of neurons obtained by analysis with Fokker-Planck equations, which corresponds to the results in Figures B and C obtained in a unit with a finite number of neurons. (E) Temporal changes in the instantaneous firing rates 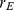 and 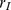. The results are similar to those in Figure B. (F) Trajectory in the (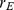, 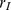) plane. A fine structure of a chaotic attractor is visible.

We constructed a network model composed of 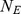 PYRs and 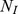 INs, each of which is modeled as a phase neuron connected to all the other neurons globally. This network might be regarded as a minicolumn [52], [58]; we refer to it as a unit hereafter. Figure 3A shows a schematic diagram of a unit. Multiple units constitute a neural network as shown in Figure 3B. One thousand PYRs are included in each unit. A typical firing pattern of a unit with 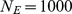 and 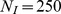 is shown in Figures 4A, 4B, and 4C. Figure 4A shows a raster plot of spikes in which high correlations exist between the firing times of different neurons. Hereafter, we refer to such firing with high correlations as synchronized firing. On the other hand, in another initial condition, asynchronous firing in which almost all neurons are silent is also observed, as shown in Figure 4D, even in the unit with the parameter values identical to those shown in Figure 4A. In other words, two firing patterns, *i.e.*, synchronized and asynchronous, coexist in the same network. Such asynchronous firing appears because the neuron model has a stable equilibrium, and it fires only when sufficiently large stimulation is applied. Synchronous and asynchronous firing patterns were the only two patterns observed for the parameters used in Figure 4.

The instantaneous firing rates 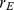 and 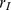 for the excitatory ensemble 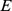 and the inhibitory ensemble 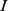 are respectively defined as
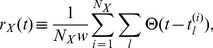
(1)

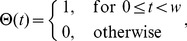
(2)where 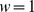, 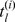 is the 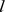
th firing time of the 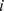
th neuron in the ensemble 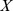, and 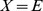 or 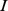. 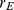 and 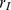 for the data in Figure 4A are shown in Figure 4B. As shown in Figure 4B, the dynamics of synchronized firing is not periodic. The trajectories in the (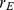, 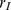) plane of the dynamics in Figure 4B is shown in Figure 4C. The dynamics of the synchronized firing in Figure 4C appear to have a complex dynamical structure.

The synchronous firing in Figure 4 can be observed only when PYRs and INs interact. Therefore, we presume that this dynamics is similar to the pyramidal-interneuron-network gamma oscillation (PING) [62]. However, the dominant periodic component in our model does not necessarily correspond to the time scale of the gamma frequency because our model is based on an abstract neuron model.

The averaged dynamics of our network in the limit of 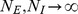 can be analyzed using the Fokker-Planck equations [52] as shown in the Methods section. By this analysis, we can calculate the theoretical dynamics of 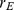 and 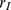 in the network with 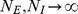. The results for the network with parameters shown in Figures 4A, 4B, and 4C are shown in Figures 4E and 4F. The structure of synchronous firing shown in Figure 4C is a strange attractor of chaos in the limit of 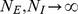, as shown in Figure 4F. Moreover, the largest Lyapunov exponent is numerically confirmed to be positive [51], which indicates that the dynamics have a sensitive dependence on initial conditions peculiar to deterministic chaos. Therefore, we refer to the synchronous firing shown in Figure 4A as chaotic synchronization. Note that the Fokker-Planck equations are deterministic partial differential equations on the probability distribution of the network in the limit of 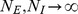 [59]. Therefore, the observed dynamics are deterministic chaos. Our previous studies indicated that chaotic synchronized firing is observed over a wide range of parameters in various models [50], [63], [64].

We regard such synchronized firing as the basic dynamics of the unit, and we further examine the dynamics in a network composed of multiple units. To reduce the computational time, below, we analyze the network dynamics using the Fokker-Planck equations.

### A Network of Multiple Units

Below, we consider a network composed of multiple units that store patterns. A schematic diagram of this network is shown in Figure 3B. 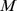 units are placed in cortical layers 2/3 of a model for the early visual cortex (V1/V2). We assume that INs in this network are 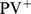 FS neurons [65]. 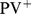 FS neurons and PYRs might be related to the generation of gamma oscillations. Cortical layers 2/3 also contain 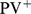 multipolar bursting INs that are related to the generation of 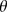 oscillations [65]. However, for simplicity we do not consider them here.

Generally, ACh decreases the magnitude of IPSCs from INs to PYRs through muscarinic 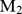 receptors [66], [67]. This effect is presynaptic, and the activities of INs themselves are not weakened by ACh. We model this effect by decreasing the strength of the inhibitory connections from INs to PYRs in layers 2/3. The GABAergic projection from the NBM is not addressed in our model. It is known that top-down Glu spike volleys are projected to layer 1 from the higher cortex and thalamic matrix circuit [68], [69]. These spikes are projected to the distal apical dendrites of PYRs whose cell bodies may exist in layers 2/3. Therefore, they are excitatory inputs to PYRs in our network. We model this effect by injecting temporal inputs to specific PYRs in the network, *i.e.*, by increasing the value of 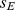 in Eq. (8) temporarily (see the Methods section). Although some theoretical research has examined the role of top-down inhibitory inputs [70], we have not included such inputs in our model.

Our network is realized by connecting multiple units as shown in Figure 3C. The connection strengths among units are determined based on previous studies [61]. As shown in Figure 3C, PYRs receive inter-unit inputs from both PYRs and INs. Conversely, INs receive inputs only from PYRs. Each ensemble of PYRs or INs has recurrent connections within the same ensemble. We define the connection strengths 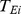 and 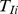 of the input to the PYRs and INs in the 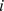
th unit [61] as
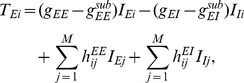
(3)

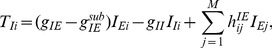
(4)where 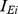 and 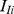 are the sums of postsynaptic currents of the 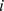
th unit defined by Eq. (10) in the Methods section. The inter-unit connections 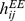, 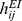, and 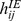 and the constants 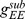, 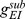, and 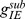 are defined based on the modified Hebbian rule as shown in the Methods section. With this configuration of connections, we store 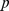 patterns in our network.

Although the number of units can be chosen arbitrarily in principle, we fix the number of units and the number of patterns as 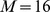 and 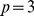, respectively, in order to perform a clear analysis. Three patterns 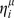 (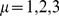) are defined as
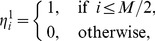
(5)

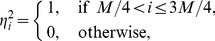
(6)

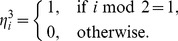
(7)


### An Ongoing State of the Network (Transitive Dynamics Among Quasi-attractors)

**Figure 5:**
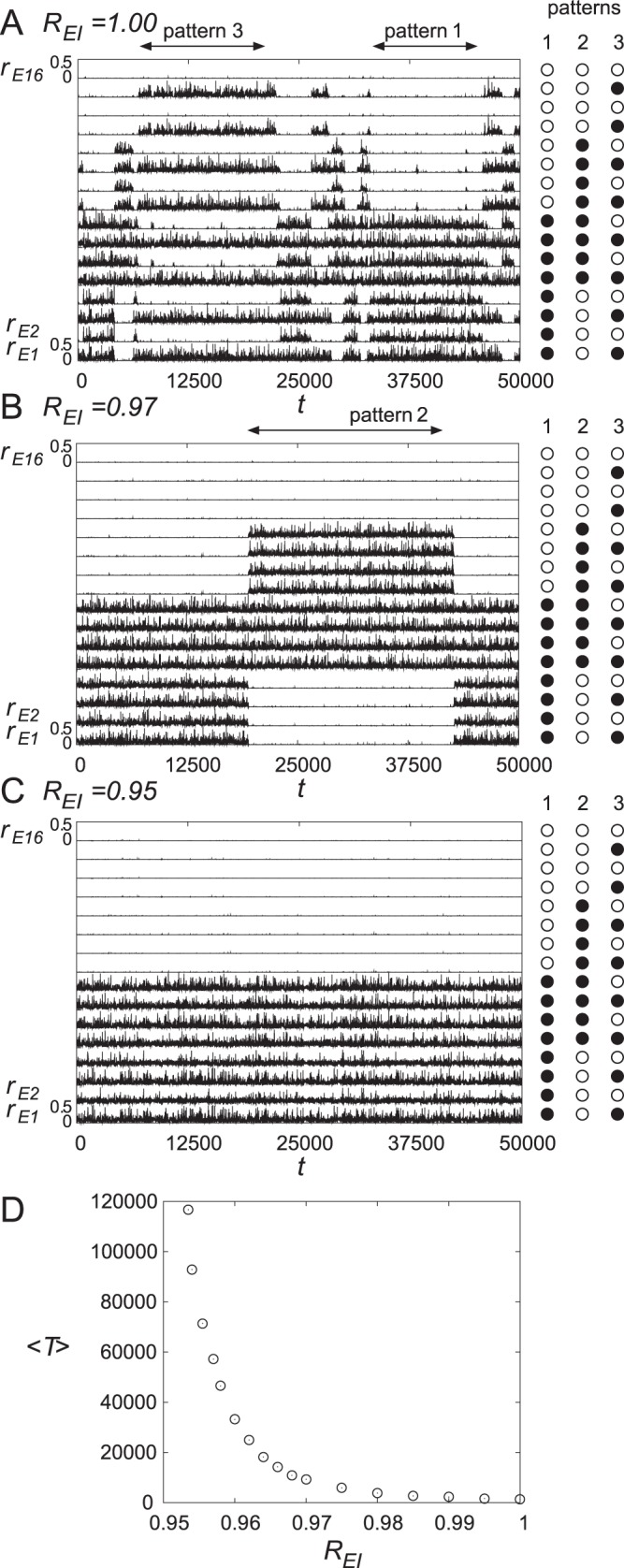
Effect of ACh in the network. (A) An ongoing state of the network of 16 units. The instantaneous firing rates of excitatory ensembles in 16 units are shown. Transitive dynamics among quasi-attractors are observed for 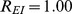, where 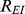 is the cholinergic reduction of inhibition that reduces the strengths of inhibitory connections as 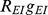 and 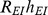 with 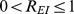. These dynamics correspond to the schematic diagram in Figures 2A and 2D. (B) When 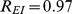, the staying time at each quasi-attractor increases. This corresponds to Figures 2B and 2E. (C) When 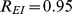, all the quasi-attractors are stabilized. Therefore, transitions between memories do not take place. This corresponds to Figures 2C and 2F. (D) Dependence of averaged staying time 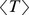 at a quasi-attractor on 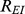. The state with 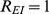 is the ongoing state and, for 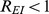, the strengths of inhibitory connections are decreased by the cholinergic reduction of inhibition. When the quasi-attractors exist, 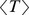 takes finite values. 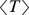 diverges at 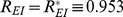, and each quasi-attractor is stabilized to become an attractor for 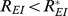.

The typical dynamics observed in a network of 16 units is shown in Figure 5A. 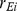 is the instantaneous firing rate of the 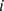
th excitatory ensemble. The transitive dynamics among three memorized patterns are observed, and these dynamics are chaotic [61]. We call such dynamics the ongoing state of this network. Moreover, we refer to the unstable memory patterns as quasi-attractors. Note that the data in Figure 5A is obtained by numerically integrating the deterministic Fokker-Planck equations. Therefore, the transitive dynamics observed in Figure 5A are not caused by noise but are deterministic.

### Stabilization of Quasi-attractors by Release of ACh to Cortical Layers 2/3

As stated above, the release of ACh onto cortical layers 2/3 decreases the magnitude of IPSCs from the 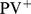 FS neurons to PYRs through muscarinic 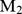 receptors. To incorporate this effect, we decrease the strength of inhibition from INs to PYRs (see Figures 3A and 3C). We decrease all 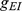 and 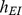 in 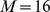 units because we assume that ACh diffuses in layers 2/3 and decreases the inhibition of PYRs through the aforementioned presynaptic, muscarinic mechanism. Here, we replace 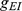 and 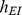 with 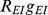 and 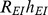 with 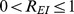, respectively, and we call 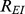 the cholinergic reduction of inhibition.

The ongoing dynamics in this network, shown in Figure 5A, are realized when 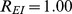. The dynamics in the network with 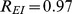 and 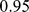, which correspond to situations in which ACh is released to layer 2/3, are shown in Figures 5B and 5C, respectively. The staying time at each quasi-attractor increases when 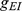 and 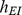 are reduced (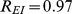). When 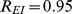, all of the quasi-attractors are stabilized. We confirmed that the stabilized attractors themselves are chaotic attractors rather than equilibrium or periodic attractors by numerically calculating the Lyapunov spectra [61] (see fine fluctuations of the instantaneous firing rates 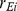). The dependence of the averaged staying time 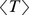 at a quasi-attractor on 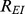 is shown in Figure 5D. It is observed that 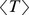 diverges at 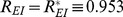, and each quasi-attractor is stabilized for 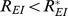.

### Selection of Quasi-attractors by Top-down Glu Spike Volleys to Cortical Layer 1

Next, we examine the effect of Glu spike volleys projected to cortical layer 1. To incorporate this effect, we changed the value of the parameter 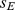 of PYRs in some units. We replace 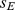 of the 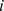
th unit with 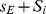, where 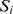 represents the effect of Glu spike volleys. Assuming that the effect of the Glu spike volleys is localized, we set 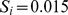 only for 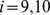 and we set 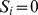 for the other units. Moreover, this input is synaptic, and therefore, the effective time of 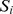 was set to be smaller than that of ACh.

As stated above, the top-down Glu spike volleys to cortical layer 1 do not deform the attractor landscape but make the trajectory jump to a quasi-attractor specified by the input in the phase space. Cortically projecting ACh is transiently released from NBM in an instant of attention [30], [40], [71], even attention to internal representations [31]–[33]. Note that this is one of the central hypotheses in this paper. According to this hypothesis, we also inject ACh and stabilize the quasi-attractor at the time when the top-down Glu spike volleys reach layer 1.

**Figure 6:**
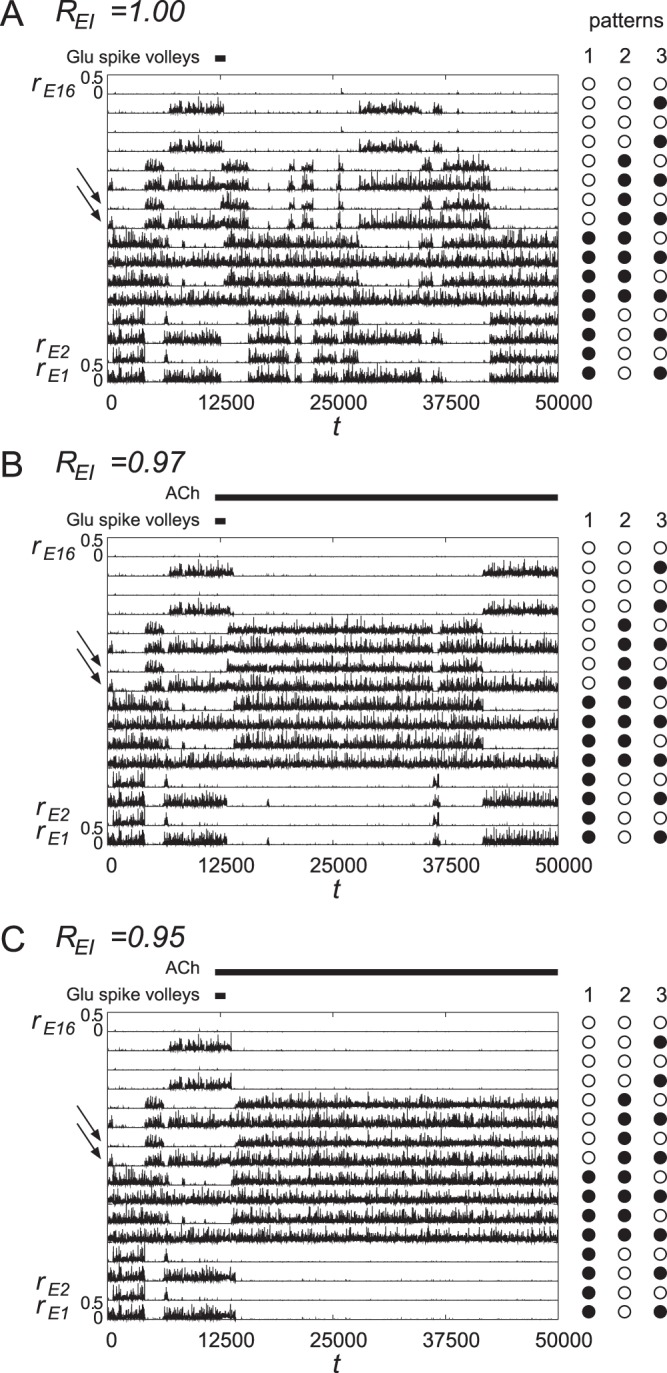
The effect of top-down Glu spike volleys to the apical distal dendrites as well as the release of ACh in cortical layer 1. The top-down Glu spike volleys are projected only to the 9th and 10th units for 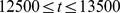. Note that both the 9th and 10th units, indicated by two arrows in each figure, are active only in pattern 2. Moreover, 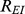 decreases for 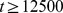 associated with the release of ACh. (A) In the ongoing state with 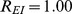, after the injection of top-down Glu spike volleys at 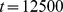, the network temporarily retrieves pattern 2. However, the retrieved pattern in the network soon transits to pattern 1 at around 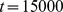 because pattern 2 is a quasi-attractor. (B) For 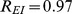, the trajectory also moves to pattern 2 but the staying time increases because of the effect of ACh. (C) In the network with 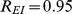, each quasi-attractor is stabilized to be an attractor. Therefore, once the trajectory transits to pattern 2, it does not move to other patterns.

Figure 6 shows the observed dynamics in our model. The top-down Glu spike volleys are projected only to the 9th and 10th units for 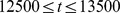, and 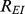 reduces for 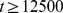 because of the release of ACh.

When we do not add ACh (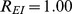) as shown in Figure 6A, the network temporarily retrieves the pattern in which the 9th and 10th units are active, *i.e.*, pattern 2 at 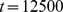. However, for 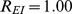, pattern 2 is a quasi-attractor, as shown in Figures 2A and 2D, and therefore, the retrieved pattern in the network soon switches to pattern 1 at around 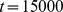. On the other hand, as shown in Figure 6B, the trajectory with 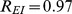 also moves to pattern 2 but the staying time increases because of the effect of ACh. This result corresponds to the case shown in Figures 2B and 2E. Finally, as shown in Figure 6C, in the network with 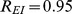, each quasi-attractor is stabilized to be an attractor, and this case corresponds to Figures 2C and 2F. Therefore, once the trajectory moves to pattern 2, it does not move to other patterns in this case.

**Figure 7:**
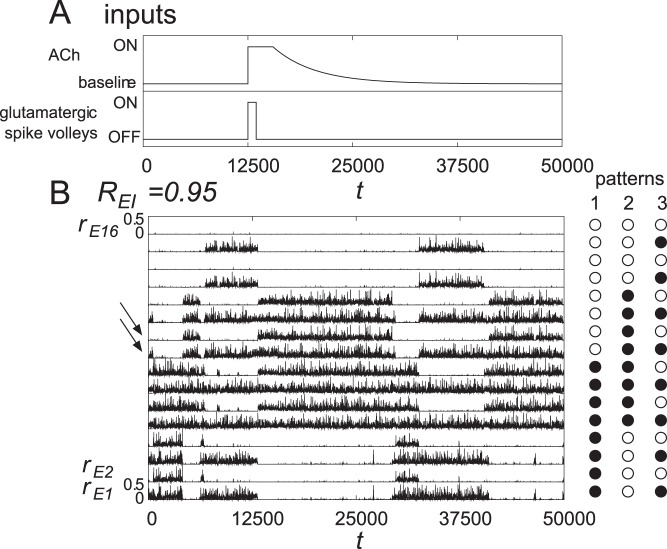
The dynamics when ACh is gradually decreased according to an exponential function. In order to simulate more physiologically plausible situations, we evaluated the network dynamics when ACh is decreased according to an exponential function. The top-down Glu spike volleys are injected to the 9th and 10th units for 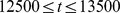. ACh is also injected at 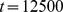. It is observed that pattern 2 is successfully retrieved while ACh is effective. Therefore, our scenario would hold as well even if the injection of ACh is temporary.

In physiological situations, the effect of ACh would gradually decay, and therefore, the complete stabilization of the quasi-attractor as shown in Figure 6C would not occur. To confirm this effect, we perform a simulation in which the concentration of ACh starts decreasing at 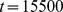 according to an exponential function, as shown in Figure 7A. Figure 7B shows that target pattern 2 is successfully retrieved persists for some time.

In summary, the top-down Glu spike volleys to cortical layer 1 make the trajectory jump to any specified pattern. However, these signals do not change the attractor landscape itself, *i.e.*, the stability of each quasi-attractor. The stabilization of each quasi-attractor is realized by the release of ACh to cortical layers 2/3.

## Discussion

### Deformation of the Attractor Landscape and Jump of Trajectories among Attractors

We observed that the ongoing state exhibits transitive dynamics between several quasi-attractors. These trajectories result from spontaneous evolution of the network state, and move from one quasi-attractor to another in the quasi-attractor landscape. Such dynamics are indicated by red arrows in Figure 2. The stabilization of quasi-attractors by the release of ACh causes a deformation of the landscape. It is emphasized that if the network is viewed as a dynamical system, the ACh concentration plays the role of a bifurcation parameter and not that of an external force applied to the system. The relatively slow, even transient functioning of ACh operates as a slowly changing bifurcation parameter (see Figure 2). By contrast, Glu spike volleys onto cortical layer 1 are an external force originating from higher cortical regions. Glu spike volleys cause the trajectory jump into the basin of an attractor, as indicated by the green arrows in Figure 2. These two processes may have different time constants. Even if the release of ACh is localized and phasic, its effects on cortical neurons persist for some time. In fact, the decrease of IPSC continues for at least several minutes after activation of muscarinic receptors by muscarine or oxotremorine *in vitro* [66], [67]. On the other hand, the duration of the Glu spike volleys to cortical layer 1 would be much shorter.

When ACh, but no top-down Glu was released, the question arises as to which attractor the state would converge to. If the state were inside the basin of a (quasi-)attractor, say A, at the instant when ACh is released, the state would remain inside the basin of A (in almost all cases) even after the ACh release (see Figures 2A and 2C). This means that the attractor to which the trajectory converges would be determined by chance. In order to make the trajectory surely converge to the target attractor (that is, during voluntary recall and reconstruction of a certain internal state), the brain requires a mechanism to make the trajectory jump into the corresponding (quasi-)attractor by some external forces (see green arrows in Figure 2). This external force can be provided (at least in part) by the top-down Glu spike volleys to cortical layer 1. Together, these two processes provide a simple mechanism for temporal reactivation of internal states in the brain.

Our network models activity in layers 2/3 of the early visual cortex (V1/V2). Although some properties (such as the size of receptive fields) vary across cortical areas, the basic structure that determines the cortical dynamics, such as the intra- and inter-cortical anatomical connectivity, neuronal configuration, and responsiveness to cholinergic release of constituent neurons, appears homologous throughout the primary and secondary visual areas V1, V2, and V4. This homology suggests that the Q-landscape may also be observed in a higher cortex such as V4 with baseline levels of ACh. One consequence of our theory is the phenomenon of large trial-to-trial variability during ongoing activity, *i.e.*, in the absence of attention and external sensory stimuli. This variability could be considerably large, and the fluctuations of unit activity are correlated with each other among neurons separated by large distances (6–10 mm) [9]. This could reflect transitions of the network state among different quasi-attractors. Moreover, it is suggested that in the absence of attention (due, *e.g*., to anesthetization), similar large trial-to-trial variability should be observed for evoked activity under the presence of external sensory stimuli, as reported by Arieli *et al.* [10]. One study suggests that attention may reduce this variability in V4 [72]. Moreover, in the IT cortex in which visual objects are thought to be stored, there is a top-down pathway from the prefrontal cortex [73], [74], and dynamics similar to our model might be observed experimentally (see also [75]).

Switching between transitive dynamics in the ongoing state and immobile dynamics in the attentional states was also observed in a network of neurons with active dendrites by Kurashige and Câteau [76]. In their model, the transitive dynamics were stochastic and the immobile dynamics were obtained by transient global inhibition. On the other hand, in our model, the transitive dynamics were deterministic and chaotic, the attractive dynamics were obtained by ACh, and the dendrites were passive. To incorporate the effects of active dendrites [76] into our model, further theoretical and experimental research would be required.

### Controversy about Cellular Effects of ACh

Although arguments about the cellular effects of ACh are inconclusive [77] and occasionally controversial, we have performed our simulations based on recent physiological data concerning cortical superficial layers. Although cholinergic cellular effects may give distinct results [77] depending on whether the application is transient (*i.e*., phasic) or persistent (*i.e.*, tonic), we have adopted transient ACh data whenever possible. As already described, in layers 2/3, both the PYR cells and the 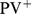 FS INs, which constitute the majority of neuron types in these layers, are essentially nonresponsive to ACh release postsynaptically [77]. However, the 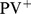 FS INs exhibit sensitive responses to ACh release presynaptically [66], [67]. We analyzed the dynamical systems consequences of these known synaptic modulations. In our model, the synaptic modulation caused by ACh is modeled by the decrease in the strengths of connections from INs to PYRs, and it decreases to approximately 95% of its original value. However, previous *in vitro* studies revealed that IPSCs decrease to approximately one-third to one-half the baseline level [66], [67]. In the following paragraphs, we will explain this discrepancy.

**Figure 8:**
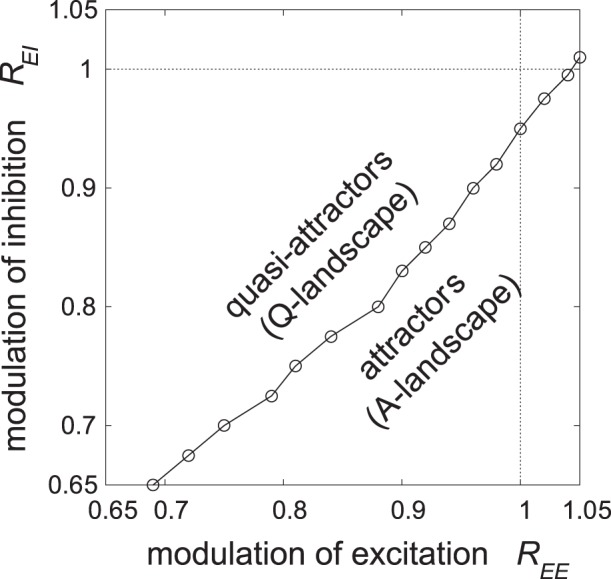
A numerically obtained boundary where quasi-attractors change to attractors as a function of cholinergic modulations 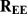 and 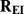. Quasi-attractors can be stabilized to attractors even when the excitatory synapses are also affected by ACh (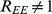). Note that the ongoing state with quasi-attractors is realized when 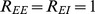. This result suggests that if at least a balance of the two opposing currents is effectively maintained, the entire scenario in the quasi-attractor hypothesis appears to hold as well.

First, we show that the range of synaptic modulations that enables our dynamics is wider than that shown in our results. Recent experimental data report that excitatory synaptic transmissions are also modulated by ACh [36], [78]–[83]. In particular, Gil *et al.* found that in *in vitro* experiments on this subject, depression of PYR to PYR connectivity occurs presynaptically under tonic (bath) applications of muscarine [78]. Therefore, we analyze the effect of the modulation of synaptic transmission between excitatory neurons. Here, we replace the strengths of excitatory synapses 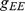 and 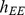 as 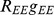 and 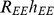 with 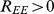, respectively, and we call 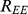 as the cholinergic modulation of excitation. By changing 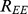 and 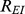 simultaneously, we numerically obtained the boundary at which quasi-attractors change to attractors, as shown in Figure 8. Note that the ongoing state with quasi-attractors is realized when 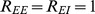. From Figure 8, the effect of change in the ACh level in Figure 7 is understood as follows. At the baseline ACh level, the cholinergic modulations are at 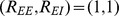. When ACh is released, 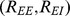 moves to 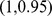, and then, it slowly relaxes to 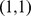. As shown in Figure 8, the quasi-attractors are stabilized to be attractors even when the excitatory synapses are slightly depressed (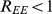), if 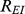 decreases sufficiently. For example, when ACh modulates both inhibition and excitation, *e.g.*, 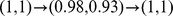, dynamics similar to those shown in Figure 7 would be observed. This result suggests that if at least a balance of the two opposing currents is effectively maintained, the entire scenario in the quasi-attractor hypothesis appears to robustly hold as well. Moreover, it is also found that our hypothesis holds when strength of inhibitory synapses is larger than 65% of its original value. When the modulations 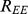 and 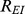 are set to be smaller, the amplitude of oscillation of 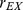 shown in Figure 5 becomes smaller, and transitive dynamics disappear.

This range of synaptic modulation is still narrower than that of physiological data (1/3 or 1/2). However, in the experimental setting [66], [67], the muscarinic receptors are activated by some amount of muscarine via puff application (as compared with bath application). As such, the activated receptors are spatially localized to a relatively small range around the puff applicator. By contrast, in our network, the strengths of all the inhibitory connections are weakened uniformly, which may better represent diffusive bath application of muscarine. A second factor that may separate our results from physiological data is that the baseline and maximal concentrations of muscarine in the *in vivo* cortex remain unknown. Given these factors, direct comparison between experiments and models remains difficult, and performing simulations with more realistic cortical neurons remains an important goal of future studies. In fact, in layers 2/3, it is known that multipolar bursting neurons also exist in addition to the fast spiking INs used in our model. These cells are reported to be related to the generation of 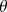 oscillations [65], although their effect has not yet fully been examined. Moreover, it is known that ACh to layer 1 depolarizes calretinin positive (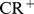) INs in layer 1 (see Figure 1) through nicotinic receptors [84], the role of which is also still under investigation.

### Possibility of Testable Predictions by the Model

Here we present testable predictions derived from our results. The results in this study can be summarized by the following two statements. First, one of the roles of cortical ACh is switching landscapes in the state (phase) space between the Q-landscape and the A-landscape. Second, the role of attractor selection is carried out by (at least in part) Glu spike volleys projected on layer 1. In the experiments with ongoing activity (*i.e.*, without Glu spike volleys and external stimulus), large trial-to-trial variability is observed in neural activity patterns, and such patterns have large correlations in space and time [9]–[11]. It is also known that such large variability is reduced under the influence of attention [72]. In our terminology, this variability and its reduction might be related to the Q-landscape of the system and its deformation into the A-landscape, respectively. In such a view, changes of the ACh level through manipulation of the cholinergic system [71] (*i.e.*, by decreasing 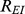 in Figure 5D) and the staying time in a quasi-attractor would have a relationship as shown in Figure 5D. Moreover, the decrease of 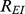 as well as 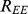 would be caused by presynaptic effects through muscarinic receptors [66], [67]. Therefore, this change of staying time in a quasi-attractor would also be observed by using agonist or antagonist of the muscarinic receptors in layers 2/3.

To test the above predictions, manipulation of ACh would be required, and it is a future problem as well as a limitation of the present work.

### Future Problems

In Figure 8, we found that our hypothesis still holds true even when ACh also modulates excitatory synaptic transmission. Viewed from a dynamical systems standpoint, what would be the implications of these effects? An intuitive argument based on attractor network theory in the classical neural network [85], [86] would be that if both excitatory and inhibitory connections in recurrent networks are all proportionally weakened, the result is that the global energy level reduces accordingly and the depth of basins becomes shallower. Meanwhile, the global structure of the landscape is expected to be qualitatively conserved. This would mean that regardless of whether attractors or quasi-attractors are involved, attentional ACh would decrease both 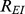 and 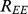 functions to make the wall between the basins lower. In turn, this change would make it easier for the top-down spike volley onto layer 1 to force the network state to jump into an assigned attractor basin. This might influence the perception of external signals because the network appears to be more responsive to external inputs under attention. In this regard, Hasselmo *et al.* [36] argued that based on the phenomenon of the depression of PYR to PYR connectivity together with presynaptic nicotinic facilitation of the thalamo-cortical (TC) circuitry in layer 4 [78], attentional ACh works to “switch between the TC and intracortical (IC) circuitries,” in which high ACh levels set circuit dynamics for attention and encoding, and low ACh levels set dynamics for consolidation. Yu and Dayan [37] discussed a similar switching mechanism with their theoretical model. Thus, our computational results are consistent with these previous intriguing arguments [36], [37], although the logical construction may not be identical. ACh has also been implicated in other functions, including the control of learning rate during reinforcement learning [87]. Investigating the relationship between other such roles of ACh and the dynamics exhibited by our model represents an important question for future research. It is also an important problem to incorporate learning process of forming attractors under the existence of ACh.

Motivated by the experimental study by Kenet *et al.* [12], we examined the situation in which there is no external visual input through layer 4. How does the corticopetal ACh relate to the attentional modulation of stimuli in a situation in which external visual stimuli exist? This may be more understandable within the context of the theory of biased competition [88]–[90] for object perception in natural scenes. At any given time, a visual scene includes a number of objects that compete with each other to be internally represented. Both bottom-up and top-down inputs work as biases to select some internal representations, but with distinct contexts. Bottom-up signals via V1/V2 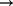 V4 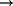 IT continually hit the orbit as external forces to virtual states in a Q-landscape, together with the top-down bias of Glu spike volleys during top-down attention. This gives ACh a chance to stabilize the landscape to an A-landscape. Without ACh release of top-down attention, such virtual states are unstable and not consciously perceived.

In the context of attractor dynamics [85], [86], attractors are often unstabilized and the dynamics become chaotic either by incorporating inhibitory neurons [16], [21] or by introducing refractory effects or self-recurrent inhibitory connections [17], [23], [24], [91] to the conventional neuron models like the analog neuron models widely used in back-propagation neural networks [92]. In our model, the stabilization of a quasi-attractor is realized when the inhibition is weakened by the release of ACh to layers 2/3, which is consistent with previous reports. It is our future problem to examine whether the quasi-attractor hypothesis proposed in this paper can provide a dynamical viewpoint for understanding nonlinear dynamics in different areas of the brain under various conditions.

## Methods

### Definition of the model

As a model of the network in layers 2/3, we define a unit of a network composed of 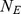 PYRs and 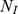 INs modeled by phase neurons defined as
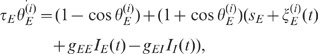
(8)

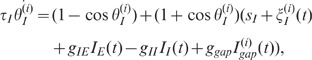
(9)

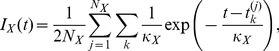
(10)

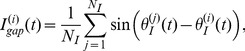
(11)

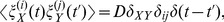
(12)which have been used previously [52]. The connections are global, and there are connections with postsynaptic currents of exponential forms among all neurons and diffusive connections among inhibitory neurons. These two types of connections model chemical synapses and electrical synapses with gap junctions, respectively. 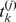 is the 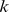
th firing time of the 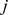
th neuron in the ensemble 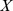 (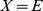 or 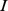), which is defined as the time at which 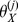 exceeds 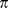. This neuron model is called the theta neuron [55], and it is also considered as a general model of a type-I spiking neuron model [56], [57]. 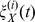 is a noise term.

### The Fokker-Planck Equations

To analyze the dynamics of a unit of a network, we use the Fokker-Planck equations, which are represented as
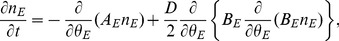
(13)

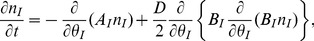
(14)

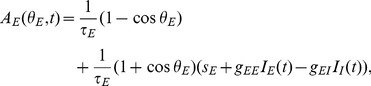
(15)

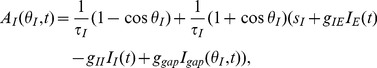
(16)

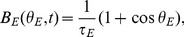
(17)

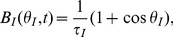
(18)

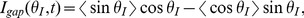
(19)

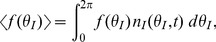
(20)for the normalized number densities of excitatory and inhibitory neurons, in which



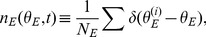
(21)

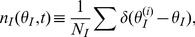
(22)in the limit of 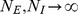 [52]. The probability flux for each assembly is defined as



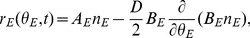
(23)

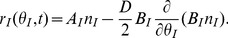
(24)


In the limit of 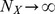, 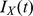 in Eq. (10) follows a differential equation written as
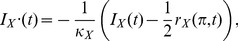
(25)where 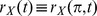 is the probability flux at 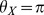.

By integrating the Fokker-Planck equations (13) and (14) and the differential equation (25) simultaneously, the dynamics of the network that is governed by Eqs. (8) and (9) can be analyzed.

### Connections among Units

The inter-unit connection strengths 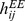, 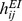, and 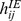 are defined based on the modified Hebbian rule as follows:
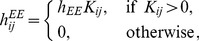
(26)

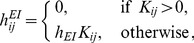
(27)

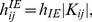
(28)

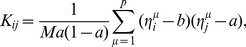
(29)where 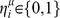 are stored patterns with firing rate 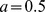, and 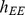, 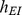, and 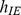 are positive parameters. In the conventional associative memory model, 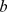 is set identical to 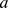 in Eq. (29); however, we use 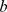 as a regulating parameter because our model has some differences from the conventional one, such as the inhibition realized by inhibitory ensembles. When 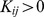, there are two types of inter-unit connections, *i.e.*, 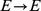 and 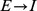, and such connections tend to induce inter-unit synchronization. On the other hand, when 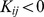, the connections 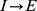 and 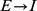 exist, and such connections tend to break the inter-unit synchronization.

Three additional parameters of regulation, 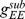, 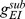, and 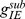, are respectively defined as 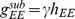, 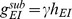, and 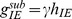 using a new parameter 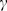 that is common to all units, and they are introduced to our model in order to keep the chaotic dynamics observed in a one-unit system with 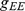, 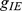, and 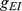 (see Figure 4). Without them, the chaotic dynamics are broken, and periodic dynamics or asynchronous firing would be observed. The roles of 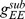, 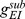, and 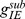 are understood as follows. Let us consider a situation in which only a single pattern is stored in the network, and we assume that 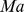 units that store the binary digit “1” in this pattern synchronize with each other. That is, they satisfy 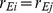 and 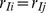, where 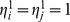. The strengths of inputs injected to such excitatory and inhibitory ensembles are calculated to be 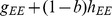 and 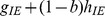, respectively, by Eqs. (26), (28), and (29). Thus, by subtracting 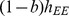 and 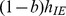 from 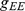 and 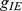, respectively, the dynamics of a one-unit system with 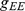 and 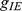 would also exist in 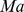 units in this network, and they tend to synchronize, *i.e.*, 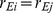 and 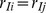, where 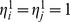 [51]. Similarly, the strength of the input injected to the excitatory ensembles that store “0″ from the inhibitory ensembles is 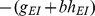. Thus, we extract 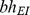 from 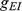. With such configurations, the chaotic dynamics observed in a one-unit system with 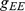, 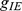, and 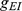 would exist in the synchronized network. The above discussion holds when the number of stored patterns is one. However, in the present network of 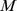 units, two or more patterns are actually stored in the network. Therefore, all 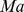 units that store the binary digit “1” do not perfectly synchronize with each other. Thus, 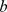 and 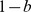 are replaced by an arbitrary constant 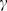, and 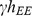, 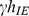, and 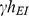 with 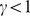 are subtracted from the connection strengths 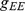, 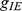, and 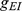, respectively.

The values of the parameters used in one unit are set close to those used in Figure 4, *i.e.*, 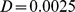, 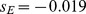, 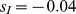, 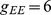, 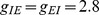, 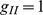, 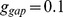, 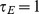, 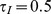, and 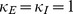. The values of 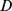, 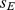, and 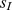 differ from the original ones in order to keep the chaotic dynamics in one unit unbroken. The values of inter-unit parameters are 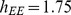, 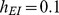, 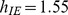, 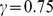, and 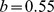.

### Calculations of Overlap and Staying Time at a Quasi-attractor

Here, we provide a method for calculating the overlap 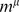 between a set of instantaneous firing rates 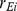 of excitatory ensembles and the stored pattern 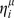 with 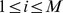. Because 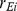 is an oscillating quantity, the overlap of the usual definition is also oscillating even when the correct pattern is retrieved. To obtain an overlap that maintains an almost constant value when the correct pattern is retrieved, we define a local peak-value function 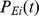. First, we define the peak time 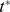 that gives a peak of 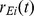, and we define the three peak times 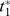, 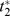, and 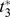 that are close to the current time 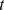, satisfying 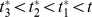. Then, we define 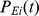 as
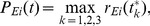
(30)


to keep the peak value for some time. Further, we transform 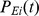 to a function 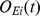 with a range of [0,1] as follows:
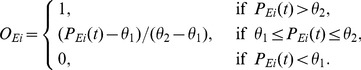
(31)By using 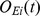, the overlap 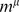 between the state of units and the stored pattern 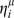 is defined as


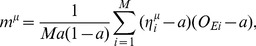
(32)


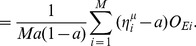
(33)

When the system stays near a quasi-attractor, the overlap of the corresponding pattern becomes close to 1. The staying time of a pattern (a quasi-attractor) is defined as the time duration during which the relationship 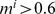 is satisfied.
